# Supplementary material for: Are carbon emissions trading and green financial instruments synergistic? -Comprehensive quantitative research based on content analysis
Source: PLoS One. 2024 Mar 7;19(3):e0298601. doi: 10.1371/journal.pone.0298601 (PMC10919601; doi:10.1371/journal.pone.0298601)
Supplement: S1 Data — (ZIP) [file pone.0298601.s001.zip › Data/地级市Z_F工作报告.zip!/░▓╗╒/░▓╟∞╩╨/└┤╫╘╣1⁄2╓┌║┼í╛┬φ┐╦╩2╛▌═°í┐_2015.docx]

政府工作报告

——2015年1月20日在安庆市第十六届人民代表大会第四次会议上

市长  魏晓明

各位代表：

现在，我代表市人民政府，向大会报告政府工作，请予审议，并请市政协委员和其他列席人员提出意见。

一、经济社会发展势头良好

刚刚过去的2014年，是全面深化改革的启动之年。全市上下在中共安庆市委的坚强领导下，深入贯彻落实党的十八大、十八届三中、四中全会和习近平总书记系列重要讲话精神，坚持稳中求进、改革创新，积极应对挑战，奋力攻坚克难，保持了经济社会持续健康发展的良好态势。预计全年地区生产总值突破1500亿元，增长9.5%左右，增幅居全省前列；固定资产投资达到1400亿元，增长18%以上；社会消费品零售总额接近600亿元，增长13%左右；进出口总额突破20亿美元，增长25.1%，增幅全省第一；城乡常住居民人均可支配收入分别增长9.5%左

右、10.5%以上，增幅分居全省第四和第二；财政收入迈上200亿台阶，完成230.8亿元，增长17.1%，增幅全省第一，非税收入占财政收入11.9%，首次降到全省平均水平以下。全年目标任务基本完成，顺利办成了一系列大事、难事和喜事，打造三个强市、建设美好安庆迈出了坚实步伐。

一年的工作和成效主要体现在六个方面：

（一）结构调整扎实有效。现代农业加快发展。启动实施“5211”工程，新增省级现代农业示范区3个、农业产业化规模以上企业71家，总量均居全省第一。耕地流转面积211万亩，流转率居全省前列。新增农民专业合作社766家、家庭农场1070家。新增国家地理标志保护产品2个，总量居全省第三。农产品加工业产值首次过千亿，居全省第二。粮食总产258.6万吨，增产4.5%。**工业经济提质增效。**“2135”行动计划付诸实施，110平方公里安庆化工新材料产业基地规划获省政府批准。环新轻量化铝缸套、泰发异辛烷、桐城新能源材料等项目建成投产，旺旺五期、飞凯超纯氧化铝、怀宁瑞沃科技产业园等项目开工，电厂二期正式核准。新开工标准化厂房558万平方米，建成273万平方米。新增中国驰名商标2件，总量居全省第二。完成工业投资750亿元以上，新增规模以上工业企业170家，规模以上工业增加值增长12.6%，增幅居全省第二。战略性新兴产业产值增长16.1%。前11个月工业利税287.7亿元，增长22.6%，分居全省第三和第二。**现代服务业稳步提升。**金融机构各项贷款余额1259.7亿元、增长17.7%，总量、增幅分居全省第三和第五。银河证券、中投证券在宜设立分支机构，场外市场挂牌企业41家。欧尚大型超市投入运营，上海新城体验式商业综合体签约落地，红星美凯龙落户枞阳。五千年文博园二期基本建成。获批创建“宽带中国”示范城市，4G网络实现乡镇全覆盖。556电商产业园成为省级示范园区，阿里巴巴·桐城产业带上线运营，安徽国际电商产业园落户岳西。电子商务交易额近20亿元，居全省前列。游客接待量3800万人次，旅游总收入增长13%。**科技创新步伐加快。**与中科院、东南大学等开展合作，建成实体研究院10家，新建院士工作站3家，人力资源产业园开园。发明专利申请量增长104.3%，专利授权量增长76.2%，增幅均居全省第一。国家级高新技术企业111家，高新技术产业增加值增长16%。

（二）城市建设管理加强。推进经济社会发展、土地利用、城乡建设、生态环保“四规合一”，完成大宜城发展战略规划和24个专项规划编制。加强老城改造、新城配套，迎宾东路、祥和路、天柱山路东段建成通车，华中西路、黄土坑东路改造完成。人民路步行街建设基本完工。东围墙街等25条背街后巷改造基本完成。新建雨水管网75.9公里、污水管网53.3公里。完成61.6公里道路绿化提升，建成游园绿地14处。完成96个老旧小区2.5万户居民天然气管网配套建设。投资25亿元建成市及8县（市）综合体育场馆，设施水平跃居全省一流。成功获批国家森林城市。加强城市管理，成功获批第三届省级文明城市，首获全国文明城市提名，省级卫生城市创建启动。城区住宅小区物业管理全覆盖工作有序推进，数字化城管系统建成。土地清理深入实施，4.5万亩批而未供、闲置和低效利用土地处置到位，得到省政府肯定，经验在全国推广。扎实推进环保创模工作，菱湖风景区水环境综合整治等重点工程全面展开，城东污水处理厂二期开工建设，安庆石化碧水蓝天工程进展顺利，脱硫脱硝率首次达到95%以上。11个大气污染防治专项行动扎实有效，淘汰黄标车和老旧车16774辆，关停超标排放企业11家，秸秆禁烧工作全省领先，空气质量优良率居全省第四。节能减排任务完成。

着眼统筹城乡发展，加快推进交通建设。宁安城际铁路建设和安庆火车站改造进展顺利，岳武高速、望东长江大桥及接线建设加快，安庆至桐城、望江、枞阳一级公路城区段建设全面启动，国省道改造完成240公里,石门湖航道疏浚基本完成，安庆新港规划即将出台，天柱山机场改造完成，将于春节前复航。

（三）改革开放深入推进。各项改革有序展开。市政府职能转变和机构改革方案获省批准实施，工商、质监、食品药品监管和地方海事（港航）体制改革完成，市农委等部门内设机构整合取得突破。实施工商注册登记制度改革，新登记企业增长47.8%。市级行政审批项目下放58项、冻结26项、取消7项，精简31.6%。工业、房地产项目审批时限分别压缩到28、29个工作日，从拿地到开工分别只要3个月、4个月，在全省最短。市政府核准投资项目由7大类减至4大类。开发园区行政事业性收费免收42项，减少收费1亿元。城区79项经营服务性收费取消7项、免收2项、降标28项，减轻企业负担2500万元。建立市级涉企收费清单制度，收费项目从252项减少到86项，每年为企业减负9300万元，成为全省收费项目最少、标准最低、监管最严的地区之一。完成市、区财政体制改革，全面实施全口径预决算。“营改增”扩大范围试点任务完成。积极跟进投融资政策变化，市级政府平台融资132亿元，超过前五年的总和，城市污水处理项目成为全国首批30个PPP试点之一。城区30个规模较小社区完成合并。农村合作金融机构全部改制为商业银行，村镇银行实现县域全覆盖。潜山、宿松农村土地承包经营权确权登记颁证试点工作基本完成。**招商引资力度加大。**成功举办北京、上海、深圳招商活动，多次赴外地开展土地推介，工作成效明显提升。嘉寓节能门窗等项目建成投产，新能源汽车产业园、润晶蓝宝石、宿松智能电子科技产业园等项目开工。引进亿元以上项目283个，其中10亿元以上项目36个，利用市外资金620亿元。**开放型经济有新发展。**实现与长三角区域通关一体化。皖西南公共型保税仓库建成运营，国际航运公司马士基在宜驻点。富士康集团入主鸿庆精机公司。安庆口岸货运量71.4万吨，增长38%。

（四）县域发展步伐加快。**工业化、城镇化同步推进。**桐城节能环保、望江纺织服装、宜秀健康食品等主导产业发展壮大，省级产业集群专业镇达到15个。县城、中心镇建设加快，潜山源潭、太湖徐桥等16个乡镇成为全国重点镇。流动人口居住证制度启动实施，城镇化率达到49.1%，提高1.3个百分点。**美好乡村建设成效明显。**中心村建设稳步推进，改造农村危房1.7万户、县乡公路268公里，加固危桥117座，乡镇农村清洁工程实现全覆盖，“三线三边”环境治理扎实有效。潜山、岳西、桐城成为全省美好乡村建设先进县，岳西县水畈村入选年度中国十大最美乡村。岳西成为国家级生态县。潜山县列入全国生态文明示范工程试点，官庄村获中国人居环境范例奖。推进精准扶贫，完成贫困村、贫困户建档立卡工作，实施172个整村推进工程，减少贫困人口12万。**水利建设不断加强。**下浒山水库施工准备工程动工，花凉亭灌区虎形潭渡槽重建工程开工。完成25个小型病险水库除险加固，解决22.7万农村人口饮水安全问题，“5588”小型水利工程建设进度全省领先。建成高标准基本农田46.8万亩，新增耕地2.3万亩。

（五）民生保障持续提升。民生支出占财政总支出的80.7%。33项民生工程全面完成。城镇新增就业9万人，下岗失业人员再就业2.9万人，城镇登记失业率控制在4.2%以内。在全省率先建立农民工工资专用账户管理机制。大病补充保险全面实施。城市低保标准达到全省平均水平，农村低保标准大幅度提升，初步建立“救急难”机制。实施“1+4”公共服务政策，城区新增社区服务机构29个、社工组织6个，采取政府购买服务方式，实施70岁以上生活困难老人居家养老服务等项目。落实单独两孩生育政策，出生人口性别比继续下降。开工保障性安居工程住房2.9万套、基本建成1.5万套，城区建成还建房109万平方米。扎实推进以船为家渔民上岸安居工程。开通城区至海口镇等6条公交线路，城区乡镇公交实现全覆盖。物价保持稳定。

（六）社会建设力度加大。节俭、圆满地承办省第十三届运动会及残疾人运动会、少数民族运动会，提振了精气神，展示了好形象，得到省委、省政府充分肯定和社会各界一致好评。国家公共文化服务体系示范区建设扎实推进，“黄金周·黄梅戏”展演活动成功举办，市文化馆新馆正式启用，市博物馆开工建设，乡镇综合文化站实现全覆盖，建成23个社区文化舞台、26个农民文化乐园。国家中小学教育质量综合评价改革实验区建设启动，完成290所义务教育学校标准化建设，新建、改扩建幼儿园29所。迎江区、大观区义务教育学校教师校长交流付诸实施。岳西、大观通过国家教育发展基本均衡县（区）验收。县级公立医院改革不断深化，药品零差率销售全面实行，宜秀区基层医疗机构“先看病后付费”做法在全省推广。我市成为全国首批养老服务业综合改革试点，新建居家养老服务站33个、农村幸福院180个，市社会福利院新院、第一光荣院和桐城“家和尊养”老年护理院投入运营。市科技馆布展升级、免费开放。全面加强信访工作，市、县两级联合接访，进京非访、赴省集体访大幅度下降。“安全安庆建设年”活动成效明显，安全事故起数、死亡人数双下降。城区油气输送管廊带隐患整治全面展开。加强社会治安综合治理，反恐维稳、打击犯罪、遏制黄赌毒等工作取得实效。开展常态化应急演练，危险化学品事故处置、森林防火应急演练成为全省示范。军地联合救援队伍建设做法得到省政府肯定，并获南京军区好评。森林防火、殡葬改革取得历史性突破。烈士陵园开工建设。村两委换届圆满完成。积极开展人民武装和“双拥”工作，国防动员和国防后备力量建设得到加强。民族宗教、外事侨务、涉台、妇女儿童、老龄、残疾人、红十字等事业进一步加强，气象、地震、防灾减灾等工作取得新进展。

过去的一年，我们深入践行群众路线，严格执行中央八项规定，贯彻落实“三严三实”要求，坚决反对“四风”，有力推动了政府工作作风转变和效能提升。自觉接受人大、政协和社会监督，办结市人大代表议案建议106件、市政协委员提案406件，办结率100%。健全政府学法制度和法律顾问制度。市政府负责同志、市直部门负责人通过“1584”热线与市民互动，每月开展电视“百姓问政”。土地、规划、城建、投融资、公共资源交易机制逐步健全，征地、拆迁、安置制度不断完善。“三公”经费支出下降22.9%。加大行政监察和审计监督力度，廉政风险防控得到加强。

各位代表、各位委员、各位同志！过去一年的成绩来之不易。这是省委、省政府和市委坚强领导的结果，是市人大、市政协和社会各界监督支持的结果，是全市人民共同奋斗的结果。在此，我代表市人民政府，向全市广大工人、农民、知识分子、干部、驻宜解放军指战员、武警消防官兵和政法干警，向各民主党派、各人民团体和社会各界人士，向关心支持安庆建设发展的中央和省驻宜单位、海内外友好人士，向在我市创业的投资者、建设者，表示崇高的敬意和衷心的感谢！

在肯定成绩的同时，我们清醒地认识到，发展中还存在不少困难和问题：经济下行压力较大，投资增长乏力，新的增长动力不足；产业层次不高的矛盾凸显，战略性新兴产业规模不大，现代服务业发展滞后；实体经济发展困难增多，信贷资金向实体经济传导不畅；公共服务水平不高，社会建设有待加强；少数干部的工作责任心、创新创造能力与新常态的要求还不适应；行政效能有待进一步提升。对此，我们一定认真对待，逐步解决。

二、全面推进三个强市建设

2015年是全面深化改革的关键之年，是全面推进依法治市的开局之年，也是完成“十二五”规划的收官之年，做好今年工作意义重大。当前，经济发展进入速度变化、结构优化、动力转化的新常态，我们要深刻认识新常态，主动适应新常态，积极引领新常态，努力实现新发展。

今年政府工作的总体要求是：全面贯彻落实党的十八大、十八届三中、四中全会，以及中央和全省经济工作会议精神，坚持稳中求进工作总基调，坚持以提高经济发展质量和效益为中心，着力做大规模与调优结构并重，着力扩大开放与深化改革并举，实施创新驱动，加强风险防控，保障改善民生，促进经济持续健康发展和社会和谐稳定，不断加快经济强市、文化强市、生态强市建设进程。

经济社会发展的主要预期目标是：地区生产总值增长8.5%以上，财政收入增长8.5%，社会消费品零售总额增长12.5%，固定资产投资增长16.5%，进出口总额增长9%，城乡常住居民人均可支配收入分别增长8.5%、9%，居民消费价格涨幅控制在3%左右，城镇登记失业率控制在4.5%以内，确保完成节能减排任务。做好“十三五”规划编制工作。

重点做好九个方面工作：

（一）进一步深化改革和扩大开放。坚持向改革要动力，以开放增活力。

**加快重点领域改革。**按照中央和省委、省政府统一部署，推进体制机制创新。**深化行政体制改革。**实行政府权力清单、责任清单、涉企收费清单制度，精简权力事项30%以上，精简市级审批项目20%以上，巩固“三最”城市创建成果。完成政府机构改革任务，积极稳妥推进事业单位分类改革。**深化财税体制改革。**统筹公共财政、政府性基金、国有资本经营、社会保险基金四大预算，完善政府预算体系，实施全面规范、透明运行的预算制度。盘活财政资金，提高管理绩效。进一步加强政府债务管理，明确偿债责任，规范举债途径，建立风险防范机制。将“营改增”范围扩大到建筑业、房地产业、金融业和生活服务业等领域。**深化国资国企改革。**完善国有资产管理体制，全面推进国有资产清产核资工作。推动国有资本逐步退出竞争性领域，实施政府平台公司分类改造。建立健全国有企业激励和约束机制，探索国有资产保值增值实现方式，防止国有资产流失。**深化投融资体制改革。**健全各级政府投融资管理体制和工作机制。积极采取PPP等模式，引入社会资本参与城市供水供气、公共交通、垃圾污水处理、重大水利工程等基础设施建设和运营。建立健全统一组织实施的重大项目投资市、县两级承担机制。**深化农村改革。**启动桐城、怀宁、望江、枞阳等县农村土地承包经营权确权登记颁证试点工作。稳妥推进农村集体产权制度改革，探索土地入股、经营权证抵押贷款等融资方式。创新农村土地承包经营权流转机制，大力发展适度规模经营，力争新增农民专业合作社500家以上、省级示范家庭农场20家以上。

**发展开放型经济。**主动参与长江经济带建设，积极对接“一带一路”战略，借鉴上海自贸区等制度创新成果，大力提升投资和贸易便利化水平。积极推进B型保税物流中心、汽车整车进口口岸等海关特殊监管区建设。支持桐城羽绒、潜山制刷产业创建省级出口示范基地。支持外贸综合服务平台建设，帮助企业拓展国际市场。大力发展加工贸易型产业。引导企业加强先进技术、关键设备、重要原材料进口。鼓励华茂等优势企业到境外建立产业园区，支持环新、白兔湖等企业并购境外研发、生产优质资源。拓展和深化与国际友好城市合作，搭建经贸、文化、教育等交流合作平台。

**拓展招商引资。**完善招商导则，着力依靠服务和环境优化招商引资。充分发挥承东启西区位、市场广阔等优势，整合多种资源招商，加大对央企、知名民企和外企招商力度。继续办好京、沪、粤招商活动，实施“千名干部驻外招商”行动。加大对发达国家产业招商力度。加强对台经贸，同步推进文化、旅游、现代农业等领域交流合作。力争引进亿元以上项目300个、10亿元以上项目50个。

（二）进一步保持经济稳定增长。发挥投资、消费、出口的拉动作用，加大对实体经济的扶持力度。

**强化实体经济政策支撑。**继续完善工业经济、现代农业、现代服务业、自主创新“四大政策”，开展政策执行效果评估，切实支持企业增加投资、扩大生产，培育新的经济增长点。继续深化“千名干部驻企帮扶”活动，及时为企业排忧解难。

**加快发展民营经济。**落实国家扶持小型、微型企业发展等政策措施。深化工商登记制度改革，降低企业创办门槛。顺应产业组织小型化、智能化、专业化的新趋势，推动资金、技术、人才等要素更多聚焦小微企业。加大财政支持力度，优化金融服务，鼓励大众创业、万众创新。

**加强中小微企业融资保障。**大力推进“信用安庆”建设。加强担保机构建设，完善银担合作、风险分担机制。深化银企对接，鼓励金融机构增加信贷投放，确保信贷增长位居全省前列。坚持服务“三农”和中小微企业定位，支持地方法人金融机构增资扩股。在支持非银行金融机构服务实体经济发展同时，加强小贷、担保、典当等行业监管，严厉打击恶意逃废债行为。引导企业突出主业，加强管理，增强融资能力。

（三）进一步加快转型升级。推进产业提升、结构优化，努力实现有质量、有效益、可持续发展。

**大力推进新型工业化。**深入实施“2135”行动计划，完成投资800亿元以上，新增规模以上工业企业200家左右。加快新能源汽车产业园建设，引导关键零部件产业集聚。深化与富士康、神华集团合作，加快智能制造和化工新材料产业发展。支持太湖功能性膜材料、怀宁电子显示材料、潜山医药、大观高档石材等产业发展。建成投运安庆电厂二期、曙光煤制氢等项目，开工华谊化工新材料、晋煤精细化工等项目，加快迎江康体娱乐创新科技园等项目建设，推进桐城、岳西抽水蓄能电站等项目前期工作。加快传统产业升级改造，支持企业并购重组。实施市经开区扩区、高新区山口片区建设，推进皖河片区开发前期工作，加快省级开发区扩区步伐。支持企业标准化厂房建设，加强产业招商，促进集约集聚发展。

**加快发展现代服务业。**大力发展金融保险、第三方物流、研发设计、科技信息等生产性服务业。继续吸引商业银行来宜设立分支机构。支持企业上市、参与股权市场交易，提高直接融资比重。实施“新三板”三年行动计划，每个县（市）新增上市企业1家以上。加强“宽带中国”示范城市建设，推进宽带向农村延伸，促进信息消费。发展电子商务，建成运营智慧产业园、长江电商生态城。鼓励建设区域快递集散中心。加快新城、百联等综合体建设，实施光彩大市场升级改造。发展教育医疗、健康养老、体育健身等产业，更好满足消费升级需求。多管齐下支持房地产业持续健康发展。

**继续加强科技创新。**深入实施自主创新“一招鲜”工程，建设技术转移中心10家、企业实体研究院20家、院士工作站5家、省级以上研发机构10家，实现科技孵化器县域全覆盖。支持发明专利申请和应用，力争发明专利申请量增幅保持全省领先。鼓励天使、风投、创投基金入驻安庆，推动设立新兴产业投资基金，扶持高新技术产业发展。积极创建中国科协海智计划安庆工作基地。新认定国家级高新技术企业50家、创新型（试点）企业10家、高新技术产品100个。

（四）进一步加强城镇建设。提升中心城市能级和城镇功能，加快新型城镇化发展。

**强化中心城市建设管理。**严格执行城市总体规划，深化区域控制性详细规划。坚持老城改造、新城配套，推进怀宁、枞阳县城与中心城市同城化发展。完成潜江路三期建设和湖心中路、湖心北路改造，开工外环北路等工程。综合整治人民路步行街周边交通问题，基本完成背街后巷改造。全面启动康熙河景观带建设。完成市一水厂异地扩建。开展全国节约集约用地模范市（县）创建活动，深化土地“三项清理”。启动全国文明城市创建工作，争创省级卫生城市。

**加强县城和中心镇建设。**开展县城规划建设管理提升行动，按照规模适度、功能完善、特色鲜明、宜居宜业的要求，高起点、高标准规划建设县城和中心镇，加强交通、水、电、路、气等公共设施建设，构建人口就近城镇化平台。加快县域经济发展，做大有较高技术含量的劳动密集型产业，促进工业化、城镇化良性互动。

**加快建设综合交通体系。**完成安庆火车站改造，确保宁安城际按期投入运营。开工建设合安九高铁，启动建设高铁枢纽站，推进阜六景、随麻安、北沿江疏港等铁路项目前期工作。建成岳武高速，加快望东长江大桥及接线、枞池长江大桥和一级公路建设，开工安庆高速出入口北移工程，推进岳武高速东沿线等项目前期工作，完成253公里国省道改造。推进安庆新港建设，落实引江济淮工程建设任务，加快江河航道升级。完成石门湖航道整治，建成凤凰作业区工业品码头。

（五）进一步推进农业现代化。加快农业发展方式转变，促进一、二、三产业融合发展。

**加快农业结构调整。**落实强农惠农政策，稳定粮食生产。深入实施“5211”工程，推动“五个百万”基地建设，扩大市场紧缺、优质特色农产品生产，打造种养加销全产业链，推行农业标准化清洁生产，走产出高效、产品安全、资源节约、环境友好的现代农业发展道路。办好上海、合肥农展。开工建设国内一流的城区农产品大市场，支持宿松新发地农批市场建设。发展特色农产品保险，支持政策性农业保险提标扩面。

**推进美好乡村建设。**完成87个中心村建设任务，改造农村危房1.3万户、县乡公路210公里，加固危桥79座。推进“三线三边”城乡环境整治，提升县城、乡镇和村庄周边环境。鼓励各县（市）区探索壮大村级集体经济新途径。加强“5588”小型水利工程建设，建成高标准基本农田25万亩，解决25万农村人口饮水安全问题。建成破罡湖闸站、虎形潭渡槽等工程，加快下浒山水库建设。

**实施精准扶贫。**加强基础设施建设，突出教育扶贫，扶持特色经济发展，加大对基本公共服务和扶贫济困支持力度。实施150个整村推进工程，启动“千家特困农户脱贫”工程，减少贫困人口10万以上。

（六）进一步加快文化强市步伐。培育和践行社会主义核心价值观，发展文化事业，壮大文化产业。

**加强公共文化服务。**全面完成公共文化服务体系示范区建设任务，确保通过国家验收。健全公共图书馆总分馆体系，推进乡镇综合文化服务中心、农民文化乐园建设。加强文物保护，完成振风塔维修工程，加快市博物馆建设。加强黄梅戏剧目创作，不断推出精品好戏。举办第七届中国（安庆）黄梅戏艺术节。开展送电影下乡、送戏进村、送文艺下基层活动。

**推动文旅融合发展。**推进皖南国际文化旅游示范区建设，加强城市形象宣传，拓展境外旅游市场。推动旅游与文化、生态、农业、体育等产业深度融合。支持五千年文博园战略重组，争创国家5A级旅游景区。扶持一批主业突出、优势明显的文化企业做大做强，推进再芬黄梅公司上市。继续实施景区品质提升工程，加强景区周边环境整治。加快城区旅游开发，加强“宜游宜秀”建设。规划建设美好乡村旅游线路，大力发展乡村旅游。

（七）进一步强化生态环保。贯彻执行新《环保法》，朝着生态优良、环境优美目标迈进。

**推进环保创模工作。**加强大气污染防治，继续深化11个专项行动，完成城区55台工业燃煤锅炉、60台茶水炉整治，确保空气质量全省领先。加强污水治理能力建设，按照全收集、全处理目标，用3年左右时间基本解决中心城市建成区水系整治、污水治理、特殊行业治污问题。完成城东污水处理厂二期、北部新城污水处理厂建设。开工建设石塘湖截污工程。加强生态县（市）创建，加强岳西国家重点生态功能区建设试点工作。整治农业面源污染，加快土壤修复，提升农村环保水平。完成百万亩森林增长提质任务。

**抓好节能减排工作。**加强重点耗能企业监管，淘汰高耗能、高污染、高排放、低效益的生产工艺。发展风能、太阳能、生物质能等新能源和可再生能源，加快天然气利用设施建设。全面完成电力、造纸、水泥等行业脱硫脱硝改造。推进建筑、交通运输、商业和公共机构等领域节能工作。

（八）进一步加强保障和改善民生。坚持守住底线、突出重点、完善制度、引导舆论，不断加强民生建设。

**切实做好民生工作。**深入实施33项民生工程。稳步提高城乡低保和农村五保保障标准。健全社会救助体系，完善“救急难”发现和救助机制。开展“工学一体”就业就学试点，实施高校毕业生就业促进计划和创业引领计划，加大就业困难人员帮扶力度。继续加强保障性安居工程和还建房建设，加快棚户区和老旧小区改造。抓好菜市场、早餐摊点和夜市排档整治提升，建成豆制品生产基地。优化城区公交线网，推进秦潭湖、红水塘公交枢纽站建设。保持物价稳定。

**大力发展社会事业。**稳步推进国家中小学教育质量综合评价改革实验区建设。加强学前教育，支持普惠制民营幼儿园发展。加快城区中小学布局调整，逐步改造农村薄弱学校，推动名校集团化发展。全面实施城区义务教育学校教师校长交流，鼓励各县（市）逐步推开。提升高中教育质量。加强职教联盟建设，深化产教融合、校企合作，加快构建现代职业教育体系。支持安庆师范学院等高等院校加快发展。全面推进公立医院综合改革，探索医疗联合体建设，在基层公立医疗机构推行“先看病后付费”做法。加快市立医院新院区建设。推进公共体育设施开放，促进全民健身广泛开展。深化国家养老服务业综合改革试点，大力推进居家养老，支持社会力量举办养老、健身、健康、医疗等服务机构。严格执行计划生育基本国策，强化出生人口性别比综合治理。巩固殡葬改革成果，推进“青山白化”治理。建成市烈士陵园。

（九）进一步强化社会治理。运用法治思维和法治方式解决矛盾和问题，努力建设全省最稳定、最安全、最和谐城市。

**深化安全安庆建设。**开展“安全安庆提升年”活动，加强生产、交通、消防安全建设，强化食品药品安全监管，严防重特大事故发生。推动安庆石化等实施油气输送管廊带迁建工程。**加强社会治安综合治理。**健全社会稳定风险评估机制，常态化开展社会面治安防控，完善“天网工程”，打击经济犯罪，维护重点工程建设秩序。全面提升应急管理及信息化水平。**做好信访维稳工作。**严格落实领导干部接访约访下访制度，依法处理矛盾纠纷，及时化解突出问题，有效防范群体性事件发生。**创新社区治理。**完善公共服务政策体系，推进非基本公共服务向社会资本开放，探索基本公共服务市场化实现方式。加强社区信息化建设和网格化管理，完成第六届社居委换届工作。健全社区矫正制度，加强人民调解工作，促进社会和谐稳定。充分发挥工会、共青团、妇联等人民团体作用。做好科普、地震、气象、档案、地方志、外事、侨务、民族、宗教、涉台、老龄、残疾人、红十字、关心下一代、无线电管理等工作。推进军民融合深度发展，支持驻宜部队建设，加强国防教育、国防动员、人民防空和民兵预备役工作，深入开展“双拥”共建活动。

三、努力建设人民满意的政府

适应经济发展新常态，加快建设法治政府，必须坚定加快发展信心，鼓足干事创业勇气，全面加强政府自身建设。

**强化担当意识。**坚决做到“心中有党、心中有民、心中有责、心中有戒”，继续发扬“拼”的精神，始终保持“干”的状态，把握发展大势，坚持问题导向，积极主动作为，奋力争先进位。不折不扣执行中央和省委、省政府的决策部署，坚定不移抓好事关安庆发展的大事难事。强化目标考核，开展跟踪问效，治理为官不为，防止懒政怠政，惩处失职渎职。

**创新工作理念。**坚持以人为本，顺应群众期待，强化为民服务，将群众满意作为最高标准，多行务实之举，多办惠民之事。增强改革勇气，弘扬创新精神，加快职能转变，把该管的事管好，把该放的权放到位，激发市场和社会活力。增强开放观念，努力开拓视野，遵循发展规律，把握宏观政策，借鉴外地经验，驾驭科学发展。

**加强依法行政。**严格依照法定权限和程序行使权力、履行职责。自觉接受人大法律监督、工作监督和政协民主监督，广泛接受社会公众和新闻舆论监督。完善公众参与、专家论证、风险评估、合法性审查、集体讨论程序，发挥法律顾问作用，规范政府重大事项决策行为。积极推行综合执法，完善行政处罚群众公议制度。深化政务公开，促进权力运行公开透明。

**不断改进作风。**践行群众路线，坚持“三严三实”，坚决反对“四风”。落实首问负责制、服务承诺制、限时办结制，办好“1584”政风行风热线，开展服务窗口群众评活动，加强政务督查，促进行政效能提升。推进政务服务体系建设，健全基层为民服务机构，方便群众办事。保持真抓实干作风，发扬钉钉子精神，持之以恒抓落实，确保干一件成一件。

**保持清正廉洁。**牢固树立纪律和规矩意识，守纪律讲规矩，做政治上的明白人。落实厉行节约规定，改革公务用车制度，严控“三公”经费和一般性支出。加强政府投资项目管理，推进公共资源交易规范化。强化行政监察和审计监督，实现公共资金、国有资产、国有资源审计全覆盖。落实党风廉政建设责任制，完善惩治和预防腐败体系，坚持有腐必惩、有贪必肃，营造风清气正的干事创业环境。

各位代表、各位委员、各位同志，形势催人奋进，目标鼓舞人心。让我们在中共安庆市委的坚强领导下，与全市人民一道，坚定信心和决心，坚持苦干加实干，为打造三个强市、建设美好安庆而努力奋斗!
